# An application of the dual identity model and active categorization to increase intercultural closeness

**DOI:** 10.3389/fpsyg.2022.705858

**Published:** 2022-09-13

**Authors:** Johanna E. Prasch, Ananta Neelim, Claus-Christian Carbon, Jan P. L. Schoormans, Janneke Blijlevens

**Affiliations:** ^1^Behavioural Business Lab, School of Economics, Finance and Marketing, RMIT University, Melbourne, VIC, Australia; ^2^Tasmanian School of Business and Economics, University of Tasmania, Sandy Bay, TAS, Australia; ^3^Department of General Psychology and Methodology, University of Bamberg, Bamberg, Germany; ^4^Bamberg Graduate School of Affective and Cognitive Sciences (BaGrACS), Bamberg, Germany; ^5^Department of Design, Organisation and Strategy, Faculty of Industrial Design Engineering, Delft University of Technology, Delft, Netherlands

**Keywords:** diversity, social inclusion, dual identity model, culture, intervention

## Abstract

The enhancement of social inclusion is a key to maintaining cohesion in society and to foster the benefits of cultural diversity. Using insights from the Dual Identity Model (DIM) with a special focus on active categorization, we develop an intervention to increase social inclusion. Our intervention encourages the participants to (re-)categorize on a superordinate level (i.e., a human identity) while being exposed to their own culture. Across a set of experiments, we test the efficacy of our intervention against control conditions on the effect of social inclusion, measured by perceived social distance. Results show an increase in cultural closeness and provide preliminary support for the use of a DIM-based intervention to increase intercultural inclusion

## Introduction

Diversity in the workplace is a double-edged sword (Milliken and Martins, 1996). On the one hand, diversity generates positive economic returns as it is linked to a larger pool of skilled people, increased innovation, problem-solving, and creativity (Cox and Blake, 1991; Williams and O’Reilly, 1998; Alesina and La Ferrara, 2005). On the other hand, diversity may also be associated with tensions and conflict (Deitch et al., 2003; Alesina and La Ferrara, 2005; Shih et al., 2013). To exploit the maximum potential of diversity, it is thus imperative that not only the downsides of diversity are reduced, but that the associated benefits are elevated (e.g., van Knippenberg et al., 2004; Homan, 2019). A theme that has emerged as a solution to foster the advantages of diversity is that of inclusion (Shore et al., 2011, 2018).

Our approach to social inclusion is inspired by Dovidio et al. (1998) proposed Dual Identification Model (DIM). The DIM suggests the use of (re-)categorization to induce a dual identification. It states that making two groups salient simultaneously—one abstract and superordinate group (e.g., a human identity), and a concrete and (sub)ordinate one (e.g., different cultures)—is a way to reduce bias between the (sub)ordinate groups. This recategorization allows people to perceive initial outgroup members as part of their new, more inclusive ingroup. In this approach it is important to maintain salience of the original identity, such that people do not feel personal identity threat when including new members to their superordinate group. It is this process that seems to make the DIM particularly useful for influencing social inclusion. Originally, the DIM has been proposed as a way to reduce prejudice and discrimination. However, the relationship of DIM with the more positive outcome of social inclusion has, to our knowledge, not been conceptualized or investigated directly.

Further, empirical studies supporting the DIM mainly rely on *passively* making both categories salient (e.g., letting participants read about the advantages of a dual identification; see Verkuyten, 2017) instead of encouraging participants to engage actively in the categorization process. Research in various areas such as learning psychology or behavior change interventions highlights the benefits of being actively involved in a task/intervention (e.g., Albarracín et al., 2005; Markant et al., 2016; Chen et al., 2018). Being actively involved is associated with higher engagement in the task/intervention and better learning, making it more likely for the intervention to be effective (Albarracín et al., 2005; Chi, 2009; Markant et al., 2016). One area that emphasizes the importance of active involvement is gamification. Gamification describes the use of game design elements to create activities that are fun, thus increasing peoples’ engagement and motivation (e.g., Deterding, 2012; Schoech et al., 2013). While gamification usually refers to the technical design of interactive games designs, elements based on the principles of gamification (e.g., active engagement, performance feedback) can be used to facilitate behavior change in other types of applications as well.

Accordingly, we aim to investigate the effects of DIM on social inclusion through active categorization, implementing elements from the area of gamification. To this end, we designed and empirically tested the Cultural Commonalities Memory Game (CCMG). This intervention is based on the DIM and utilizes elements of gamification. People are encouraged to actively (re-)categorize different cultures to achieve a dual identification, which then increases social inclusion, potentially leveraging the benefits of diversity.

### Diversity and inclusion

The terms diversity and inclusion are often used interchangeably (Shore et al., 2018). However, there are substantial differences. As Winters (2014) puts it, “perhaps the most salient distinction between diversity and inclusion is that diversity can be mandated and legislated, while inclusion stems from voluntary actions” (p.206). This distinction especially applies in a workplace setting. Governmental or organizational efforts to increase (cultural) diversity in the workplace are often successful in increasing the number of employees with varying backgrounds. However, only having a diverse workforce does not imply that organizations can foster the positive effects that are associated with it. Instead, a “focus on inclusionary practices can promote the potential advantages and opportunities of having a diverse workforce” (Shore et al., 2018). As such, the voluntary actions suggested by Winters could be the implementation of a diversity training. Diversity refers to the attributes that can differ between individuals, potentially leading to the perception that another person is different from oneself (van Knippenberg et al., 2004). In contrast, inclusion can be described as the degree to which an individual perceives that they are an esteemed member of a group they belong to through experiencing treatment that satisfies their need for belonging but at the same time makes them feel that their individuality is not only recognized but appreciated (Shore et al., 2011). Diverse work teams have access to a larger pool of knowledge, skills and abilities, and hence have more resources to work with and benefit from (Ely and Thomas, 2001). However, these resources are only fully available when the employees feel safe to share their unique identities without fear of repercussions (Shore et al., 2018). It is crucial that the individuals experience being valued and respected (Ely and Thomas, 2001) and feel safe to express their unique views (Shore et al., 2018). In an inclusive environment, diversity is appreciated as a resource and can facilitate mutual learning and growth (Shore et al., 2018). Inclusion is associated with higher job satisfaction (Acquavita et al., 2009), enhanced team engagement (Nembhard and Edmondson, 2006), as well as psychological health and work attitudes (Hitlan et al., 2006). In this way, inclusion can contribute to the overall productivity of an organization. Social inclusion occurs when people self-identify as part of a group and as individuals. People adopt their identities based on self-categorization (e.g., Tajfel and Turner, 1979). A prominent approach investigating the effects of social categorization and identification is the Social Identity Approach.

For more details on the concept of social inclusion as adopted in this paper, its measurement, and information about the differences to related concepts and their measurement, refer to Table 1. We provide this table as background because even though these concepts are similar on the surface, it is important to clearly delineate them for their application to our research so that we can identify the best measures for these concepts.

**Table 1:** Table defining and differentiating between relevant and related concepts, highlighting the relevance for the current study.

Concept	Definition & relevant background	Typical measurement	Relevance for current research	Fit^1^
Social Inclusion	Social inclusion can be defined **“as the degree to which an employee perceives that he or she is an esteemed member of the workgroup through experiencing treatment that satisfies his or her needs for belongingness and uniqueness”** (Shore et al., 2011).	Qualitative studies or questionnaires assessing the perceived inclusion of individual employees in their workgroup (e.g., Mor Barak et al., 1998, 2006; Findler et al., 2007; Acquavita et al., 2009), the perceived inclusiveness of their leader (e.g., Nembhard and Edmondson, 2006), or of their organization as a whole (e.g., Pelled et al., 1999; Roberson, 2006)	The measurement of social inclusion traditionally focuses on the perceived inclusion of employees in various areas. Instead of measuring employees’ perception of feeling included, we are interested in the effects of our intervention on the social inclusiveness of individuals toward their peers. Hence, we cannot adapt traditional social inclusion measures in our current project.	0
	Relevant areas:			
	Workgroup inclusion, leader inclusion, perceived organizational inclusion, organizational inclusive practices, inclusive climate (Shore et al., 2018)			
Psychic Distance	“**The psychic distance concept refers to the individual’s perception of the differences between the home country and the foreign country**” (Sousa and Bradley, 2008). Psychic distance is a subjective and multifaceted construct (e.g., geographical distance, legal system, and business ethics; Sousa and Bradley, 2008; Child et al., 2009; Vaccarini et al., 2017). The level of cultural distance is one of the most relevant dimensions of psychic distance (Vaccarini et al., 2017) and is often used as proxy for psychic distance (Child et al., 2009).	Questionnaires assessing the perceived *differences* between home country and a particular host country along each of the psychic distance dimensions (12 dimensions; Child et al., 2009; Vaccarini et al., 2017)	Psychic distance is a concept used in research around the trade between countries. The measures assess the perceived distance of an individual to another country or culture rather than to individuals from said culture. Our research is focused on the interindividual relationship and as such we are interested in the perceived distance between individuals from various cultures.	–
Cultural Distance or Cultural Novelty (Wang and Varma, 2019)	Cultural distance can be defined “**as the degree to which cultural values in one country are different from those in another country**” (Sousa and Bradley, 2008).	Traditionally calculated (Shenkar, 2001, 2012) using the formula developed by Kogut and Singh (1988); the formula uses information from models describing differences in cultural dimensions, such as the research by Hofstede (1980); initially differentiating between 4 cultural dimensions) or the GLOBE project (9 cultural dimensions; House et al., 2004); the formula results in a composite index illustrating the deviation between the target cultures along each of the cultural dimensions (Kogut and Singh, 1988); see for example Wang and Varma (2019)	Cultural distance “can be used to assess differences in national culture” (Sousa and Bradley, 2008) and is typically used in research centring around expatriates in specific and the cooperation between companies from different countries in general. We are not interested in the differences between cultures *per se* but in interindividual relationships and thus in the *perceived* differences/distance of a single individual toward other individuals from another culture.	––
	There is controversy about the conceptualization and measurement of this construct, which is often used as a “seemingly simple and standardized measure of cultural differences” (Shenkar, 2001).			
Psychological Distance	“**Psychological distance is a cognitive separation between the self and other instances such as persons, events, or times**” (Baltatescu, 2014). It “**is a subjective experience that something is close or far away from the self, here, and now**” (Trope and Liberman, 2010).	There is a broad array of measures for psychological distance as the chosen measurement tool typically depends on which of the for facets is targeted. In some cases, one type of distance is measured as a proxy for another type of distance (e.g., spatial distance, such as choosing a distant seat from another person, as a measure for social distance; Trope and Liberman, 2010). Other measures include questionnaires assessing the distance dimensions (e.g., Spence et al., 2012; Chen and Li, 2018) or use construal level measures as proxy for psychological distance (e.g., Chandran and Menon, 2004)	Mental representations are associated with different types of attitudes (Trope and Liberman, 2010). We are interested in the attitudes from individuals from one culture to those from another culture. Hence, investigating psychological distance through its dimension social distance, i.e., the mental representations associated with social relationships, seems fitting for our current study.	+
	The concept is defined within the Construal-Level Theory (Trope and Liberman, 2003) and encompasses the four dimensions temporal, spatial, **social**, and hypothetical distance (Trope and Liberman, 2010). Psychological distance can influence the nature of the mental representations of objects in someone’s mind (Trope and Liberman, 2010) and thus affects people’s reactions to cognitive object cognition and evaluation (Chen and Li, 2018).			
Perceived Social Distance	Perceived social distance can be defined **“as the overall level of perceived similarity between the self and a typical target group member and reflects the degree to which one perceives oneself to be generally representative of the group**” (Jones, 2004).	Questionnaires assessing participants’ perceived similarity/ familiarity/ anticipated ease of social interaction with a target group (Jones, 2004); Measurement of explicit attitudes possible	Cultural attitudes are a sensitive topic, which is why asking participants directly about their attitudes toward individuals from other cultures might be prone to social desirability effects. Hence, we decided to employ the physical distance estimations in our studies to assess implicit social closeness as a more reliable measure in this context.	+
	Perceived (social) closeness is linked to cognitive and affective processes such as familiarity and liking, and can facilitate interactions, making them more effective (Swift, 1999).	Questionnaires assessing participants’ physical distance estimations (e.g., Carbon and Leder, 2005; Carbon, 2010); Measurement of implicit attitudes possible		
Perceived Geographical Distance	In people’s minds, physical locations and the distances between them are represented in so-called cognitive maps (Tolman, 1948; Carbon and Hesslinger, 2013). These maps are subject to systematic distortions by factors such as familiarity and emotional involvement; hence, rather than being exact physical representations of the reality, physical distance estimations reflect affective and cognitive influences (Kerkman et al., 2004) and as such can be used as indicator for social closeness.	Questionnaires assessing participants’ distance estimations between relevant cities/ locations/ people (e.g., Kerkman et al., 2004; Carbon and Leder, 2005; Carbon, 2010)	We are interested in the socially inclusive attitudes of individuals toward their intercultural peers. Perceived social closeness is positively associated with emotional involvement (Ekman and Bratfisch, 1965), making it an essential antecedent for social inclusion (see Carbon, 2010; Carbon and Hesslinger, 2013). When people like or are familiar with someone or something, they feel closer to them (Swift, 1999). The perceived social closeness of the participants towards people from other cultures can be used as proxy for socially inclusive attitudes	++
		Questionnaires asking participants to compare distances between cities/ locations/ people (e.g., Ekman and Bratfisch, 1965)		

1 Fit of the concept and its measurement for our research project, from -- (poor fit) to ++ (good fit). In this table, closeness and distance are understood as the two poles of a continuum, describing the same construct from different points of view.

### Social identification

The Social Identity Approach combines insights from Social Identity Theory (Tajfel and Turner, 1979) and Social categorization Theory (Turner et al., 1987). The basic premise of the Social Identity Approach is that people categorize themselves and others in social categories. Social categorization works as an activator for different levels of self-concepts and identities (Tajfel and Turner, 1979). Categories influence how people see themselves and others (Tajfel and Turner, 1979), they work as a filter for thoughts, responses and behavior (Nicholls and Rice, 2017).

Categorization is an automatic psychological reaction to being confronted with different stimuli and leads to forming groups on different levels of abstraction (Mervis and Rosch, 1981). The more abstract the category, the more inclusive it is: While concrete categorization focuses on details, abstract categorization targets the overall picture. In a social setting, the most abstract or superordinate category can be associated with a human identity, the ordinate category with a social identity, and the most concrete or subordinate category with a personal identity (Turner et al., 1987).

Basing their research on the Optimal Distinctiveness Theory (ODT; Brewer, 1991), Pickett et al. (2002) suggested that “social identities serve the function of satisfying individuals’ need for assimilation (ingroup inclusion) and their need for differentiation (distinctiveness from others)” (p. 543). In other words, individuals identify with those social groups that allow their needs for belonging and uniqueness to be met in a given situation (Shore et al., 2011). This notion highlights the role of individual-needs in group processes (for an empirical review summarizing the consequences of the ODT for social identification, refer to Leonardelli et al., 2010).

The literature suggests that social categorization itself is enough to create group effects such as ingroup bias (Tajfel and Turner, 1979), where people value ingroup members more than outgroup members (e.g., Spears, 2007) and are more empathetic in response to the needs of fellow ingroup members (Tarrant et al., 2009; Montalan et al., 2012). Traditionally, ingroup bias is associated with negative effects such as prejudice and discrimination toward the outgroup. However, ingroup bias also leads to more favorable treatment of ingroup members. This ingroup favoritism might even be more influential on preferences and associated behavior than outgroup derogation (Feld et al., 2016). The DIM aims to extend this ingroup favoritism to the former outgroup and thereby seems especially suitable to induce social inclusion.

### Dual identity model

The Dual Identity Model (DIM; Dovidio et al., 1998) suggests that encouraging individuals to identify themselves and others on two levels of abstraction simultaneously can facilitate intergroup relations. Encouraging individuals to (re-)categorize at a superordinate level aims to (re-)introduce the former outgroup members as part of a new, more inclusive ingroup and therefore promotes the idea of sameness among these group members at the superordinate level. Categorising someone as an ingroup member extends trust to fellow ingroup members (Schwegler, 2009) and accentuates the similarity to them (Turner and Reynolds, 2001). According to Trope et al. (2007), similarity to other individuals (in this case, ingroup members) is a form of social closeness. Feeling similar to someone is linked to a perception of closeness and connection. Ingroup categorization and social closeness are both associated with empathy, liking, and trust (e.g., Swift, 1999; Hogg and Terry, 2000; Trope et al., 2007; Brewer, 2008).

However, even though the potential of re-categorising on a superordinate level to reduce ingroup bias has been supported by several studies (Gaertner et al., 1989; Dovidio et al., 1998; González and Brown, 2006), blurring or even dissolving the already existing group boundaries can threaten someone’s own current social identity and induce perceived identity threat (Hornsey and Hogg, 2000a). A threat to the social identity can be a possible loss of status, the absence of the possibility to improve low status, a poorly defined or unclear ingroup prototype, or indistinct intergroup boundaries (Hornsey and Hogg, 2000a). Perceived identity threat can lead to efforts to strengthen the intergroup boundaries through cultural prejudice and discrimination (Hornsey and Hogg, 2000a). Hence, when people are encouraged to give up their original identity for a more inclusionary superordinate group, they might perceive the undermining of their personal identity as a threat that can then lead to efforts to strengthen the intergroup boundaries and could increase bias rather than reduce it.

The DIM suggests keeping the original, subordinate group salient through the re-categorization process so as to prevent perceived identity threat (Dovidio et al., 1998; Hornsey and Hogg, 2000a). The induced dual identification can lead to a state in which both identities (i.e., the subordinate and the superordinate identity) are acknowledged and promoted simultaneously (Hornsey and Hogg, 2000a).

Even though Haslam et al. (2003) proposed the DIM as a way to reduce diversity issues in general, Dovidio et al. (2013) suggested that dual identification might be particularly beneficial in intercultural contexts: People’s cultural identity is a fundamental aspect of their self-concepts and self-esteem, which makes it unlikely for the cultural identity to be readily abandoned. Therefore, keeping the cultural identity salient is especially important in intercultural contexts.

Empirical research on the DIM has mainly focused on reducing the negative effects of ingroup bias. For instance, Hornsey and Hogg (2000c) showed that participants primed with a dual identity showed lower explicit ingroup bias than participants primed with either a concrete or an abstract group, and Horton and Griffin (2017) report that a single identification in the workplace is associated with higher conflict than a dual identification (or an even more complex pattern of identity). However, as discussed previously, reducing the negative effects of ingroup bias does not directly help to foster the benefits of diversity; instead, an environment of social inclusion needs to be the goal (Thomas and Ely, 1996; Shore et al., 2011). People feel included when their needs for belonging and uniqueness are met, and the DIM highlights the importance of being part of a group While appreciating the differences. In this research, we focus on the potential benefits of DIM in the context of social inclusion.

Empirical studies supporting the DIM mainly rely on passively making both identities salient (e.g., Hornsey and Hogg, 2000b,c; Glasford and Dovidio, 2011; Scheepers et al., 2014; Charnysh et al., 2015; Verkuyten, 2017). However, literature in various areas of research (e.g., learning psychology, cognitive psychology [memory], gamification, health psychology, and social marketing) suggests that being actively involved in either interventions or learning tasks is beneficial for the desired outcome (e.g., Albarracín et al., 2005; Markant et al., 2016; Chen et al., 2018). This is especially notable in the context of this paper, as the frequent use of certain categories can enhance the accessibility of these categories in someone’s mind (Hornsey, 2008), making it more likely for these categories to be used.

### Active categorization

Being active during a task is not only associated with higher engagement (Chi, 2009) but is also perceived as valuable by the participants (Chen et al., 2018) and can make it more likely for an intervention to have the desired impact (Albarracín et al., 2005). Active participation can produce stronger and longer-lasting changes in attitudes and behavior in comparison with passive participation (Schou, 1985; Kvam, 2000; Veenhof et al., 2006). We believe that an intervention encouraging *voluntary* active participation might be especially beneficial in the context of sensitive topics, such as social inclusion or cultural ingroup bias because forcing people to take part in such interventions can lead to resistance to the information (Stone and Moskowitz, 2011), and thus can make those interventions pointless or even produce backlash.

One approach that emphasizes the importance of being actively involved is gamification. Gamification uses game-design elements to develop activities which are fun (e.g., Muntean, 2011; Deterding, 2012; Schoech et al., 2013), thereby increasing the participant’s motivation and engagement in the activity (e.g., Muntean, 2011; Schoech et al., 2013). Successfully engaging people in a task is vital for the effectiveness of that task, independent of whether the goal is to collect data or to encourage behavior changes (Lumsden et al., 2016). One key gamification principle is that a game has to be challenging, including the possibility of failure (Schoech et al., 2013). Losing or gaining points according to the performance shown in the game and receiving feedback about the achievements is a key mechanism to increase people’s motivation (e.g., Deterding, 2012; Schoech et al., 2013).

Based on these insights, we developed a gamified intervention that encourages participants to actively categorize on a superordinate level (human identity) While being exposed to a subordinate level (their own cultural identity): The Cultural Commonalities Memory Game (CCMG).

### Our approach: The cultural commonalities memory game

**Figure 1:**
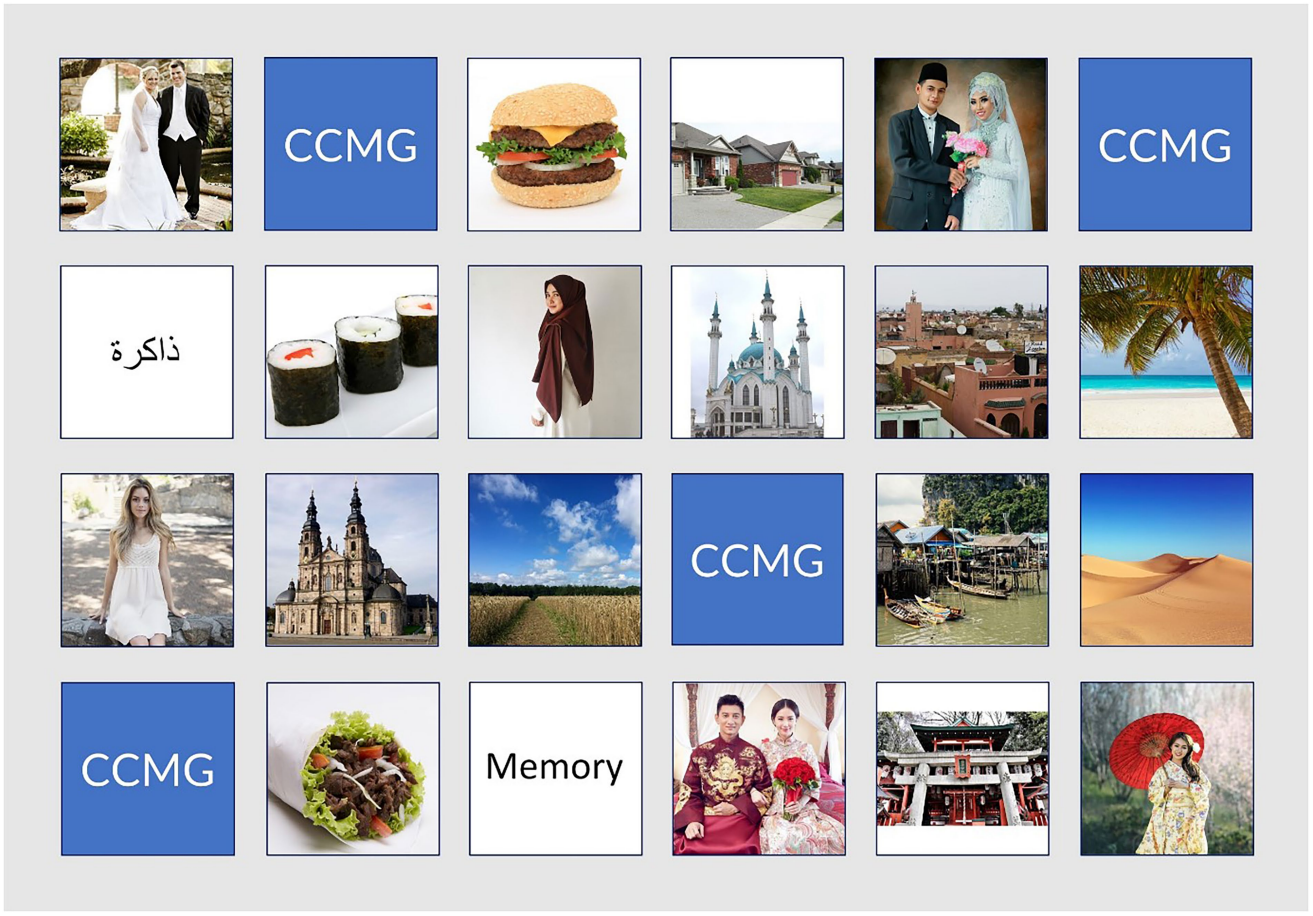
Example interface of the online CCMG. The different images indicate the uncovered matches and the blue tiles with CCMG-symbols indicate (at that given point) non-uncovered/face down items. The *script* match is not fully uncovered yet. All the images included in the CCMG were obtained from public databases, namely www.pixabay.com and www.pexels.com.

The CCMG (see Figure 1) works mostly like the popular children’s game *Memory*. Memory typically consists of 24 cards with images from different themes (e.g., animals, flowers) including 12 identical pairs. Cards are laid out randomly and face down. The first player chooses two cards to turn over. If they match, the cards are removed and the player scores one point. If they do not match, the cards are turned back over, and the next player’s turn is up. To win, players must turn over the cards and remember the locations of particular images in order to secure a match. Memory can be played by one or more players using a physical card set or *via* a computer interface.^1^

We adapted the setup and the rules of Memory to our research purposes in creating the CCMG, inspired by the game design by Emami (2007). Specifically, we developed an online version of this game in the context of cultures. While our game was inspired by existing multicultural matching games,^2^ the CCMG is different in two important aspects. First, the CCMG requires matching cards depicting similar, not identical, images belonging to a superordinate category. This was inspired by the game concept of Emami (2007), designed for a Dutch multicultural context. For example, burger, kebab, and sushi belong to the category of savory dishes; church, mosque, and temple belong to the category houses of prayer. Second, the CCMG requires the matching of three rather than two cards to avoid making only the differences between the ordinate groups salient. For example, using only a doughnut and a mooncake could encourage the participants to focus on differences between them. The inclusion of a third image such as a baklava should lead the participants to recognize the differences on a concrete level and to search for similarities on an abstract level, i.e., deserts. Hence, the CCMG requires participants to categorize on a superordinate level (a human identity) While being exposed to their own culture as subordinate level category (e.g., the Western culture, the Middle Eastern culture, etc.).^3^

The aim of the game is to find the matches as fast and errorless as possible. Performance scores are presented after finishing the game. These scores indicate the error rate (number of tries and number of cards flipped) as a measure for accuracy and the time spent on the memory as a measure for speed. When playing the game multiple times, the participants can improve their scores, adding an additional gamification element to the game. The scores illustrate the performance and highlight achievements. In this way, they work as extrinsic and as intrinsic motivators, facilitating the experience of powerful psychological processes like self-efficacy, feelings of mastery, achievement and autonomy (Bandura, 1977; Deterding, 2012).

To test the effectiveness of the CCMG, we conducted a pre-test and two experimental studies. All experiments were approved by the Ethics Advisory Network of the first author’s university. The goal of the pre-test was to investigate the success of the CCMG to encourage participants to categorize on a more abstract level (leading to a superordinate identification) relative to a control game (the control game only includes images from one culture that belong together to more concrete categories, leading to a subordinate identification). In the two experimental studies, we aimed to investigate the effects of the CCMG on social inclusion and ingroup bias as assessed with various measures, and tested for possible underlying mechanisms, namely construal level (i.e., abstraction level of thinking) and awareness of ingroup bias. For more information about the general procedure of maximizing the statistical power of our experiments and the inclusiveness of our samples, refer to Appendix A.

## Pre-test

In this pre-test, we investigated if playing the CCMG encouraged participants to categorize on a more abstract level in contrast to playing the control game as an indicator for a superordinate categorization induced by the CCMG. According to literature, categorising on a more (vs. less) abstract level induces a more (vs. less) abstract level of construal (i.e., level of thinking; Ülkümen et al., 2010). We conducted a between-subjects design experiment with the two memory games (CCMG vs. control) as independent variable and the Kimchi-Palmer-Figures task (Kimchi and Palmer, 1982) and an abstraction task more thematically related to the task at hand (described below) as dependent variables.

### Method

#### Participants and Sampling

We conducted an *a priori* power analysis to calculate the needed *N* of participants; as we have directed hypotheses (i.e., one-tailed) and are interested in testing the null hypothesis, we set the α-level to 0.05, a 1-β-(power) level to 0.95 and a medium effect size of Cohen’s *d* = 0.5. We chose a medium effect size for the power analysis because we designed our intervention to have real-world impact. As such, we were not focused on any effects smaller than a medium-sized effect. The power calculation indicated we needed an *N* = 176. Accordingly, we distributed an online questionnaire through Qualtrics to recruit a sample that follows quotas for age, gender, and education level following their distribution in the US population based on US Census data. We designed our intervention to be played by individuals from all different types of cultural backgrounds, which is why we did not select the participants according to their background or assess this information in this early phase of evaluating the intervention. We oversampled and collected data from *N* = 212 participants to account for data quality. Excluding participants who did not take the experiment seriously before analyzing results (e.g., random letters or numbers as answers), we ran the analyses with *N* = 189 participants, resulting in a 1-β-(power) level of 0.96 (*M_age_* = 41.85, SD*_age_* = 13.96, *n* = 98 women, see Table 2 for frequencies on educational level). The participants received a small incentive for participation (Qualtrics offers the reward that best suits the specific survey project and that is most likely to appeal to a diverse community of participants).

**Table 2:** Frequency of educational levels for pre-study, Study 1 and Study 2 provided in percentages.

Educational level	Primary school	Secondary school	State college or university	Private college or university	Community college	Institute of technology		
Pretest	4.8	22.8	37.6	19.6	11.1	4.2		
Study 1	2.9	28.1	26.9	25.7	11.1	5.3		
	**Less than high school**	**High school graduate**	**Technical qualification/Certificate**	**Bachelor’s degree**	**Honor’s degree**	**Master’s degree**	**Doctorate**	**Advance Diploma/Diploma**
Study 2	0.7	21.5	20.2	24.6	3.7	14.5	1	13.8

#### Material

##### Control game

To examine if playing the CCMG encouraged participants to categorize on an abstract level, we designed a ‘control’ game. In contrast to the CCMG, the images used in the control game belong together in a more concrete category (e.g., three burgers; three churches), making only one culture (i.e., the Western culture) salient. After finishing the game, performance scores are presented, namely the error rate (number of tries and number of cards flipped) as a measure for accuracy and the time spent on the memory as a measure for speed.

##### Level of categorization

To determine the level of categorization induced in the participants by the CCMG versus the control game, we presented two tasks. First, we included the commonly used Kimchi-Palmer-Figure task (Kimchi and Palmer, 1982; for an example figure and more information about the calculation of the scores used as a dependent variable, refer to Appendix B), assessing the overall abstraction level of participants’ construal level. As an additional measure and one that is more closely related to the memory game, we included, what we call, the ‘abstraction task’. Similar to the *broad* versus *narrow categorization* task (Isen and Daubman, 1984), participants are asked to create categories that are grouping given items together. Those categories differ in their level of abstraction and depend on participants’ current thinking style. We asked the participants to label the categories they identified While playing the games. We analyzed the labels of the categories by dividing them into four groups of different levels of abstraction. The classification was discussed until agreement was reached. Each label was attributed to a number between 1 (i.e., very concrete) and 4 (i.e., very abstract; see Appendix C for the categories and associated labels). Afterward, we calculated the sum of the abstraction of those category labels for each participant.

#### Procedure

After completing a set of demographic questions, participants were randomly assigned to either play the CCMG or the control game twice (in addition to a practice round). After each round, participants were asked to type in their scores (time played, number of tries, number of cards flipped). This was to ensure that the participants actually played the games and for us to be able to exclude participants with unrealistic scores as determined by a pilot. After having played the games three times, the participants were asked to complete the Kimchi-Palmer-Figures task and the abstraction task. The individual Kimchi-Palmer figures were presented randomly. At the end of the experiment, we presented a free text entry box for potential feedback about the study. The study took approximately 15 min to complete.

### Results

#### Hypothesis testing

First, we tested the effect of the game played (CCMG vs. control) on the level of abstraction as measured by the abstraction task. Using a t-test with 10,000 bootstrap samples with the different games as independent variable and the abstraction level of the used categories in the abstraction task as dependent variable, we found that participants playing the CCMG used significantly more abstract categories While playing the game in contrast to participants playing the control game, *t*(183) = −17.63, *p_one-tailed_* < 0.001, 95% CI [−9.607; −7.715], *M_control_* = 16.17, SD*_control_* = 3.11, *M_abstract_* = 24.83, SD*_CCMG_* = 3.55, *d_Cohen_* = 2.595.

However, when we ran the same analysis with the Kimchi-Palmer-Figures task as dependent variable, we did not find a significant difference between the two games (*p* > 0.05, 95% CI [−0.123; 0.082]). Notably, the abstraction task and the Kimchi-Palmer-Figures task did not correlate significantly with each other [*r* = 0.046, *p* > 0.05, 95% CI (−0.106; 0.190)].

#### Participant’s feedback

The voluntary feedback from our participants indicated that they found playing the games fun. In our sample, 39.7% of participants used either the words fun or funny and/or words related to fun to describe the games, without us explicitly asking for their experience.

### Discussion

Our analyses indicate that playing the CCMG encourages participants to use more abstract categories relative to playing the control game. However, the more abstract categorization during the game did not lead to a generally more abstract level of construal. This might be due to the presence of the lower-level categories (i.e., the three different cultures) leading to the induction of a dual identification instead of to just a higher-level identification. As mentioned before, categorization is a context-specific process (Hogg and Terry, 2000; Hornsey, 2008), which might be why we only see the effects of our CCMG in a task relevant to cultures.

Participants indicated that playing the games was fun, although we did not explicitly ask about this variable. The fun factor is an important finding because the gamification literature suggests that fun increases participants’ motivation (e.g., Muntean, 2011; Schoech et al., 2013), hence facilitating behavior change (Lumsden et al., 2016) and could potentially contribute to long-term commitment to play the game. Successfully engaging people in a task is vital for the effectiveness of that task (Lumsden et al., 2016). Hence, it is beneficial for the effectiveness of our game that it is fun to play.

We can conclude that the CCMG seems to work as intended as playing the CCMG encouraged participants to use categories on a more abstract level. Therefore, we took the CCMG (and the control game) forward to use in our next study.

## Study 1

In this study, we investigate the effects of playing the CCMG on social inclusion. Additionally, we test whether the games are fun to play - and if the two games are comparable in the amount of fun they evoke. Finally, we want to investigate the role of construal level further.

According to Swift (1999), perceived (social) closeness is linked to affective and cognitive processes such as liking and familiarity, and can make interactions easier and more effective. Moreover, closeness is positively associated with emotional involvement (Ekman and Bratfisch, 1965), and as such is an essential antecedent for social inclusion (see Carbon, 2010; Carbon and Hesslinger, 2013). Hence, we measure perceived closeness as a proxy for social inclusion (refer to Table 1).

Our pre-test suggests that playing the CCMG does not directly influence the overall construal; nevertheless, we wanted to investigate if the construal level acted as mediator between the games and social closeness. The Kimchi-Palmer-Figures task appears to measure construal level on a very general level and might not be relatable to our manipulation itself, which focuses on (social) re-Categorization. We included a construal level measure based on the categorization of images as a measure more closely related to our games. Such a measure allowed us to explore whether the Kimchi-Palmer-Figures task was not an appropriate measure of construal level in this context or if the CCMG simply does not influence the overall construal level directly.

Moreover, our pre-test indicates that playing the games is fun. Gamification literature suggests that perceived fun in an activity can increase participants’ engagement and motivation, which in turn can influence the effectiveness of said task (e.g., Muntean, 2011; Schoech et al., 2013; Lumsden et al., 2016). Hence, it is important that the games do not differ in the amount of fun they evoke, because that could lead to one game being more effective in the manipulation than the other one, which would present a confound in the interpretation of our results. Only if the games are comparably fun, we can assure that DIM drives the potential effects of the games and not fun.

Based on the literature and the pre-test, we developed the following hypotheses we tested in Study 1.

*Hypothesis 1*: Playing the CCMG increases social closeness compared to the control game.

*Hypothesis 2*: Playing the games is equally fun for both games.

To test our hypotheses, we conducted an experiment which utilized a between-subjects design with the two memory games (CCMG vs. control) as the independent variable, social closeness and fun as dependent variables, and construal level as mediator.

### Method

#### Participants

The procedure of data collection was identical to that of the pre-test, except as noted below. We collected data of *N* = 208 participants. We excluded participants who did not take the survey seriously before analyzing the data (e.g., unrealistic scores concerning their performance in the memory (realistic scores based on pilot testing: 50–350, 15–80, 30–300), including random numbers or letters in the grouping task and/or the manipulation task, >50% same answers to the 15 individual distance estimations or values <100 and > 50,000, letters or words in the distance task) and extreme outliers in the distance task [>3*IQR (interquartile range)]. We conducted the analyses with 170 participants, resulting in a 1-β-(power) level of 0.95 (*M_age_* = 41.78, SD*_age_* = 15.78, *n* = 88 women, *n* = 1 ‘*I would rather not say*’).

#### Material

As in the pre-test, we included the CCMG, the control game, the Kimchi-Palmer-Figures task and the abstraction task (here used as a manipulation check). We used a distance estimation task to assess social closeness as our main dependent variable and a grouping task to measure construal level. The new tasks are described below.

##### Distance estimation task

One way to measure social closeness is the assessment of physical distance estimations (refer to Table 1). Physical locations and the distances between them are represented in so-called cognitive maps in people’s minds (Tolman, 1948; Carbon and Hesslinger, 2013). Cognitive maps are systematically distorted by factors such as familiarity and emotional involvement, which is why physical distance estimations, rather than being exact physical representations of the reality, reflect affective and cognitive influences and as such can be used as indicator for social closeness. As Kerkman et al. (2004) stated: “On the surface, estimating the physical locations of places would appear to be a purely cognitive task concerning physical space, but the evidence indicates that it is strongly associated with social attitudes, and may be caused by them” (p. 268). This idea has, for example, been used by Carbon and colleagues (e.g., Carbon and Leder, 2005; Carbon, 2010; Carbon and Hesslinger, 2013). These authors asked participants to estimate physical distances between cities (as the crow flies) to assess psychological closeness and corresponding attitudes toward political decisions or other countries and cultures. For instance, Carbon and Hesslinger (2013) found that positive attitudes toward leading politicians was associated with smaller distance estimations toward a country, indicating social closeness. Hence, to test the effects of the CCMG on social closeness, we measured perceived closeness using the measure adopted from Carbon and Leder (2005). The participants are presented with all the possible distances between two cities each from the cultures included in the CCMG, namely the Western culture (here represented by the USA to account for our US-sample, with Los Angeles and New York City), the Middle Eastern culture (Kabul and Baghdad) and the East Asian culture (Beijing and HongKong). In line with the procedure by Carbon and colleagues (Carbon, 2010; Carbon and Hesslinger, 2013), the participants are asked to *estimate* all the resulting 15 distances (as crow flies) between city pairs. This implies that each participant elicits distance measures for cities *across* cultures (e.g., Los Angeles and Kabul) and *within* a culture (e.g., Kabul and Baghdad). For all the 15 city pairs, refer to Appendix D.

##### Grouping task

As an alternative construal level measure more closely related to the CCMG, we added a grouping task adapted from Liberman et al. (2002). In the original task, participants are presented with lists of items that need to be categorised in groups; depending on the construal level, participants use more or fewer groups (few groups would indicate a more abstract level of construal than many groups, as more abstract groups are more inclusive than concrete groups). We adapted the task to relate closely to our CCMG. The participants were asked to categorize images, half of them already known from the games, half of them new, into as many groups as they deemed appropriate (for an overview of all images, refer to Appendix E). The number of groups generated was used as dependent variable. As in the original task, the more groups participants would use, the more concrete their level of construal would be.

#### Procedure

The procedure follows the pre-test, except as noted here. After playing the games, participants rated how much fun they had playing the game on a 7-point Likert scale from 1 = *not at all* to 7 = *a lot*. We then presented the distance estimation task and asked participants to estimate the distances between six cities associated with the cultures included in the CCMG. The pairs of cities were presented randomly. Following the distance task, construal level was measured through Kimchi-Palmer figures (Kimchi and Palmer, 1982; Appendix B), the grouping task (see Appendix E), and the manipulation check ‘abstraction task’. The study took approximately 20 min to complete.

### Results

Note that the following analyses were run with 10,000 bootstrap samples unless otherwise noted.

#### Manipulation check

Validating the results of our pre-test, the manipulation check ‘abstraction task’ (two-sample *t*-test) showed that participants used more abstract labels to describe the matches in the CCMG than to label the matches in the control game, *t*(154) = −17.26, *p* < 0.001, 95% CI [−8.793; −6.994]; *M_control_* = 16.29, SD*_control_* = 2.81; *M_CCMG_* = 24.19, SD*_CCMG_* = 2.91; *d_Cohen_* = 2.765.

#### Hypothesis testing

##### Hypothesis 1: Playing the CCMG increases social closeness

To test whether playing the CCMG increases social closeness to other cultures, we ran a mixed linear model with random intercepts and random slopes for the within-subject manipulation distance type (distances between cities within – e.g., New York City to Los Angeles – vs. across cultures – e.g., New York City to Kabul). The square root of the distance estimations (15 individual estimations per participant) was used as the dependent variable and the games (CCMG vs. control game) and the distance type as the two independent variables.

We did not find any significant interaction effects between the two independent variables (*p* > 0.05, 95% CI [−0.49; 4.96]) meaning that any differences found between the games were not influenced by distance type. We are interested in the unconditional main effects, which is why we decided to respecify and simplify our model as much as possible. Hence, we ran the analyses to investigate the main effects of the independent variables game and then for distance type.

In line with our hypothesis, we find that the distance estimations were significantly smaller after playing the CCMG than after playing the control game [*M_control_* = 4,324.93, SD*_control_* = 2,630.26; *M_CCMG_* = 4,250.83, SD*_CCMG_* = 2,706.89; *Cov_distancetype_* = 80.08 (refer to Lorah, 2018)]. We also find that the distance estimations within one country/region were significantly smaller than the distance estimations across countries/regions (*M_within_* = 1,844.90, SD*_within_* = 1,303.86; *M_across_* = 4,897.28, SD*_across_* = 2,573.20). See Table 3 for an overview of the results. We can conclude that playing the CCMG significantly increased social closeness.

**Table 3:** Summary of linear mixed model analyses for the predictors game and distance type.

Model summary		−2LL(6) = 21,925.82				
Predictors	*F*	*β*	*SE*	95% CI		value of *p*
				Lower	Upper	
Game condition	−1.43	−0.095	0.759	−3.034	−0.055	0.037
Distance type	26.84	0.011	0.693	25.490	28.197	< 0.001
**Estimates of covariance parameters**						
Intercept Variance	42.44	17.756	7.681	45.218	75.383	< 0.001
Distance type Variance	80.08	23.633	10.906	82.687	125.415	< 0.001

CI: confidence interval; −2LL: −2 log-likelihood.

##### Hypothesis 2: Playing the games is equally fun for both games

The results of a one-sample *t*-test with fun as the dependent variable and a test value of 5 indicated that the games are indeed fun to play, *t*(169) = 3.49, *p* = 0.001, 95% CI [0.171, 0.629], *M* = 5.41, SD = 1.54, *d_Cohen_* = 0.266. A Bayesian two-sample *t*-test with an uninformative prior (default setting in IBM SPSS Statistics 26) revealed a Bayes Factor of *BF_01_* = 6.693, indicating moderate evidence for the null hypothesis that the games evoke similar levels of fun (*M_control_* = 5.49, SD*_control_* = 1.47, *M_CCMG_* = 5.33, SD*_CCMG_* = 1.61).

##### Other observations

We aimed to control for the covariates age, gender, and political orientation. To do this, we ran robustness tests. We found that the results of the analyses while controlling for the covariates did not differ qualitatively from the analyses not controlling for the covariates. Hence, the above-described results are reported without controlling for the covariates (see Simmons et al., 2011).

To assess the influence of the games on construal level in a more general way, we examined the effect of playing the CCMG on the Kimchi-Palmer-Figures task and the grouping task (two-sample *t*-tests, the independent variable games and the dependent variables Kimchi-Palmer-Figures task and grouping task). We find that the games did not influence the construal level as tested by our measures (*p* > 0.10).

Furthermore, we ran a mediation analyses (Process; Hayes, 2016) with games as independent variable, social closeness as dependent variable, and construal level as mediator. The analysis with either of the two construal level measures showed that the construal level did not mediate the effect of the CCMG on social closeness (5,000 bootstrap samples; *p* > 0.10).

### Discussion

Measuring the effects of an intervention on social inclusion or inclusiveness as perceived by an individual is challenging. To measure social inclusion directly, experiments should conduct group testings over a period of time, wherein participants could actually experience being included in the group. While such a study would be a great way to investigate the effects of our intervention, as a first step we decided to adopt a less resource-intensive approach (refer to Table 1). In this current study, we target the measurement of antecedents of social inclusion — more specifically, social closeness. This study presents first results supporting our claim that dual identification encouraged through active categorization can bring cultures closer together. The distance estimations after playing the CCMG were significantly smaller than after playing the control game, which indicates a greater cultural closeness.

Our pre-study indicates that playing the games is fun. According to gamification literature, the fun experienced during a task is linked to increases in participants’ engagement and motivation and can hence facilitate behavior change. This means activities that differ in fun would also differ in the level of engagement and motivation they evoke in participants and hence result in different amounts of behavior change. Therefore, it is important that the games do not differ in the amount of fun they evoke — which seems to be the case. Hence, this intervention to social closeness is a fun way to enhance interactions in a multicultural setting.

However, the increased social closeness is not mediated by changes in construal level. This might be due to the exposure to the original level while categorising on the superordinate level. Our game aims to induce a dual categorization on two levels of abstraction instead of inducing a single higher-level identification. Even though participants actively categorize on a more abstract level While playing the CCMG compared to the control game, they are made aware of their more concrete cultural identity simultaneously which may cancel out the effect of abstract level categorization on construal level.

An alternative explanation for the effects of the CCMG could be the mere exposure effect. The mere exposure effect describes a positive relation between familiarity and positive attitudes (Zajonc, 1968, 2001). This means, just presenting players with images associated with other cultures might increase their positive attitudes toward these cultures – independent from any specific identity pattern. To rule out this alternative explanation, we decided to design a second control game for our further studies.

Traditionally, the DIM is set in a context of ingroup bias and discrimination. Even though we specifically designed our intervention to increase social inclusion, we wanted to investigate if the CCMG does also influence these more negative consequences of group identification in the form of bias and discrimination. Moreover, the literature suggests that people need to be aware of their biases to be able to modify their associated responses (Chartrand, 2005). Many cultural interventions are aimed at creating awareness of one’s biases so that the participants can then actively modify their behaviour. It could be the case that the CCMG helps players to become more aware of their inherent cultural bias through playing the CCMG, for example as they may attempt to first try and match cards from only one culture together before realizing that they should match images of different cultures. Based on these insights and to validate and expand the results of Study 1, we conducted a second study.

## Study 2

The aim of Study 2 was to validate the findings of Study 1 with a different group of participants and to expand them by including a second control game to investigate the alternative explanation that the effects of the CCMG are based solely on exposure rather than dual identification, resulting in a total of three different intervention games. We did not find any relationship between the CCMG and construal level in Study 1, which is why we wanted to investigate the role of bias awareness as an underlying mechanism explaining the effects of the CCMG instead. To validate our game with a sample from a different Western country, we selected the Australian population as our target sample. As we included participants from the US population in the pre-test and Study 1 irrespective of their culture, these first experiments indicate that the CCMG can work for a population as a whole, independent from the different cultural backgrounds represented in that country. However, as the attitudes and behavior from members of the cultural majority toward the cultural minority are typically in the focus of cultural diversity research, we decided to specifically focus on this very relationship in this last experiment, selecting Caucasian Australians as participants. In line with the traditional context of the DIM, we aimed to explore the effects of the CCMG on explicit attitudes, namely cultural bias and diversity perceptions as indicator for explicit social inclusion. To make our results more robust, we included several covariates known to influence ingroup bias in this current experiment. To increase the chances of finding any existing effects, we implemented cultural priming techniques to increase participants’ awareness of their own cultural background in half of our sample. We test the following hypotheses in Study 2.

*Hypothesis 1*: Playing the CCMG increases social inclusion.

*Hypothesis 2*: Awareness of the ingroup bias mediates the effects of the CCMG on explicit social inclusion.

*Hypothesis 3*: Playing the CCMG decreases explicit cultural bias.

*Hypothesis 4*: Awareness of the ingroup bias mediates the effects of the CCMG on explicit cultural bias.

### Method

#### Participants

The procedure of data collection was identical to that of the pre-test and Study 1, except as noted below. We conducted an *a priori* power analysis to calculate the needed N of participants; for this 3 (games) × 2 (priming) between subjects-experiment with directed hypotheses, we set the α-level to 0.10, the 1-β (power)-level to 0.90 and an expected medium effect size of *f* = 0.25. The power calculation indicated we needed an *N* = 287. We oversampled and collected data from *N* = 300 participants to account for data quality issues. Further, we believe that active categorization to induce a dual identification might be especially beneficial in the workplace, which is why we targeted a working population.

Before running the analyses, we excluded participants who indicated that they did not want to be included in the analysis and those who did not take the survey seriously. We used the same exclusion criteria as in Study 1. Additionally, we excluded participants who did not read the questions properly, with special consideration of reverse coded questions (excluding participants who flatlined on the dependent variables). We conducted the analyses with *N* = 297 participants, resulting in a 1-β-(power) level of 0.91 (*M_age_* = 38.94, SD*_age_* = 13.81; gender distribution based on self-identification: *n* = 164 female, *n* = 129 male, *n* = 3 *Gender Variant/Non-conforming*, *n* = 1 transgender male).

#### Material

##### Exposure game

To investigate if the effects of our CCMG are based solely on the exposure effect rather than dual identification, we designed a version of the memory game that includes cards depicting images associated with the three target cultures but does not require the players to categorize on a superordinate level. Instead, the player needs to find matches of three cards that are associated with just one of the three cultures (e.g., three mosques, three plates of sushi). In that way, the players are exposed to the three different cultures but are not encouraged to identify on two different levels of abstraction.

##### Priming cultural background

Literature suggests that increased salience of the cultural group is associated with higher ingroup bias and thus that priming can help to investigate the effects related to cultural ingroup bias (e.g., Chen et al., 2014). To make sure the priming indeed increased the salience of people’s cultural background, we decided to include three different priming tasks in our study, namely asking the participants to (1) indicate their cultural background, (2) answer a questionnaire about (cultural) group identification (two subscales of the Collective Self-Esteem scale (CSE) by Luhtanen and Crocker (1992), and (3) write about an experience linked to their cultural background.

To control for the effects of priming, we included a non-priming condition that was structured the same way as the priming condition. Participants were asked three questions about themselves that were unrelated to their cultural background (e.g., ‘Do you wear glasses or contact lenses?’), answered the two subscales *past focus* and *future focus* of the Temporal Focus Scale (TFS; Shipp et al., 2009; example item: ‘I think about what my future has in store.’), and wrote an essay about their last grocery shopping experience. We decided to include the non-priming condition to account for potential contextual factors under which our intervention might work; if the priming leads to different results, this would have consequences for real-world application. All the experimental conditions were randomized over the priming conditions. For more information about the exact tasks, refer to Appendix F.

##### Dependent variables

To measure social inclusion, we presented the distance measure from Study 1, adapted for the Australian context. We replaced the US-American cities with two cities located in Australia, namely Sydney and Perth (example distance: Sydney - Beijing; see Appendix G). Our distance measure assesses implicit attitudes; however, prior studies investigating the effects of DIM usually include measures for explicit attitudes, which is why we included the personal dimension of the Diversity Perception Scale (DPS) by Mor Barak et al. (1998; example item: ‘I think that diverse viewpoints add value’; see Appendix H) as a measure for explicit inclusion. Diversity orientation does not only indicate if individuals are open to include people from diverse backgrounds but is also linked to social closeness as measured by distance estimations (e.g., see Kerkman et al., 2004), making this measure fitting for out study.

To test the effect of the games on explicit bias measures, we included several stereotype and attitude measures toward Asians and Arabs. Because cultural bias is context-sensitive (Pedersen et al., 2000; Forrest and Dunn, 2007), we decided to include questionnaires specifically developed for the Australian context, namely the Attitudes Against Asians Scale (AAsS; Walker, 1994; example item: ‘I would not like an Asian to be my boss.’ see Appendix I) and the Attitudes toward Muslim Australians (ATMA; Griffiths and Pedersen, 2009; example item: ‘All Arabs are potentially terrorists.’; see Appendix J). Additionally, we decided to include the expressed preferences in line with Axt (2017). In his paper, he demonstrates that ‘the best way to measure explicit racial attitudes is to ask about them’ (Axt, 2017). Even though Axt conducted his research in the USA, the nature of the expressed preferences is so general that we decided to use it anyway, adapted to our context. Participants were asked to indicate their cultural Expressed Preferences toward Asians or Arabs (ExpAsians; ExpArabs) on a 7-point Likert scale from-3 = *I strongly prefer Asian/Arabic Australians to European Australians*, *via* 0 = *I like European Australians and Asian/Arabic Australians equally*, to +3 = *I strongly prefer European Australians to Asian/Arabic Australians*.

Awareness was operationalized by two items, ‘Do you think you are biased?’ and ‘Did playing this game make you more aware of your bias?’. We administered both questions on a 7-point Likert-scale from *strongly agree* to *strongly disagree*.

##### Covariates

Similar to Study 1, we included common covariates and tested whether they influenced our results. For more information about the included covariates and their measurement, refer to Appendix K.

Because the topic of cultural bias is highly sensitive and most of our measures are very direct, we included a social desirability measure, namely the Social Desirability Scale (SDS; Stöber, 2001; example item: ‘In traffic I am always polite and considerate of others.’ see Appendix L), to control for possible social desirability effects.

#### Procedure

After answering some demographic questions to ensure the distribution of our sample on these variables represents their distribution in the Australian population based on Australian Census data and to assess some potential covariates (age, gender, education level), we primed cultural background to induce a higher (cultural) group identification. We randomly assigned the participants to the priming-group or the non-priming control group. Following that, all participants were randomly assigned to play either the CCMG, the control game, or the exposure game twice (plus a practice round). After each round, participants were asked to insert their scores in the corresponding boxes of the survey to ensure the participants actually played the games and to be able to exclude participants with unrealistic scores (as determined by pre-tests).

Because social inclusion is our main dependent variable, we first presented the measures assessing social inclusion (distance task and DPS), followed by the tools measuring explicit cultural ingroup bias (AAsS, ATMA, ExpAsians, ExpArabs). Within those two blocks (social inclusion and cultural bias), the tasks and the questions within the tasks/questionnaires were presented randomly to avoid possible sequence effects. Following this, we asked the participants two questions about their *awareness* of their bias, namely if they thought that they were biased and if the memory game increased the awareness of their own cultural bias. After the awareness questions, the non-priming group was presented with the CSE (as this group had not answered the CSE up to that point) and the priming group was presented with the TFS as filler questionnaire.

Following, all participants were asked to indicate with which political party they identify most (Liberal Party, Labour Party, Greens), and to fill in measures for self-esteem (Rosenberg Self-Esteem Scale; RSES) and mood (Mood Short Form; MSF). As the last measure, we included the SDS to control for possible social desirability effects. The study took on average 30 min to complete.

### Results

#### Data preparation

We used the mean scores of each questionnaire as dependent variable or covariate score, respectively. To make results across scales comparable, we z-transformed all variables for the hypotheses testing. Prior to that, we ran principal component analyses (PCAs) to check for the appropriateness of the scales (DPS, ATMA, AAsS, MSF, RSES, SDS). Additionally, we calculated Cohen’s alpha as a reliability check for the questionnaires. For more information about these analyses, refer to Appendix M. Note that the internal consistency of the DPS was poor (α_DPS_ = 0.587), and thus it imperative to interpret the results from the DPS with caution.

As a robustness check, we ran all the hypothesis tests two times, with or without the potential covariates age, gender, education level, political orientation, mood, and self-esteem, and the control variable social desirability. We consider the results as robust, as the analyses controlling for the covariates and the control variable were qualitatively similar to the ones reported here. Note that the following analyses were run with 10,000 bootstrap samples unless noted otherwise.

Prior to the hypothesis tests, we explored the relationships between the dependent variables. Results for the social distance measure as our main dependent variable showed small to no correlations to the explicit bias and social inclusion measures, which is important to note specifically in light of the results we present below. For more details about this analysis, refer to Appendix N.

#### Hypotheses testing

##### Hypothesis 1: Playing the CCMG increases social inclusion

To test if playing the CCMG (compared to the control and the exposure game) increases social inclusion, we implemented two measures, namely the distance task and the DPS.

To investigate if playing the CCMG increases closeness between the three different cultures, following a model comparison, we ran a mixed linear model with random intercepts and random slopes for the within-subject manipulation distance type (within a culture vs. across cultures), the square root of the distance estimations as dependent variable, and the priming condition and the three different games as between-subject manipulations. We did not find any three-way or two-way interaction effects between the three independent variables (*p* > 0.05), so we ran the same analysis again without interaction terms to investigate the main effects of the independent variables game, distance type, and priming condition on the distance estimations.

In line with our hypothesis, we find that the distance estimations were significantly smaller after playing the CCMG than after playing the control game or the exposure game [*M_control_* = 5,915.96, SD*_control_* = 3,441.89; *M_exposure_* = 5,820.51, SD*_exposure_* = 3,254.12; *M_CCMG_* = 5,661.52, SD*_CCMG_* = 3,284.44; *Cov_distancetype_* = 55.75 (refer to Lorah, 2018)]. Additionally, the same analysis showed that the distance estimations within one country/region were significantly smaller than the distance estimations across countries/regions (*M_within_* = 2,755.39, SD*_within_* = 1,984.81; *M_across_* = 6,569.63, SD*_across_* = 3,169.89). The distance estimations did not differ significantly between the non-priming and priming group (*M_non-priming_* = 5,729.40, SD*_non-priming_* = 3,380.24; *M_priming_* = 6,305.69, SD*_priming_* = 3,297.96). See Table 4 for an overview of the analyses.

**Table 4:** Summary of linear mixed model analyses for the predictors game, distance type and priming.

Model summary		−2LL(8) = 35,267.47				
Predictors	*F*	*β*	*SE*	95% CI		value of *p*
				Lower	Upper	
Control vs. CCMG	2.01	0.105	0.789	0.578	3.690	0.007
Exposure vs. CCMG	1.76	0.139	0.814	0.301	3.503	0.019
Distance type	29.30	−0.004	0.607	28.110	30.480	< 0.001
Priming	1.26	0.013	0.658	−0.086	2.552	0.034
**Estimates of covariance parameters**						
Intercept Variance	95.05	18.568	8.016	98.094	129.792	< 0.001
Distance type Variance	55.75	42.268	10.330	78.420	119.181	< 0.001

CI: confidence interval; −2LL: −2 log-likelihood.

To investigate if playing the CCMG increases social inclusion as measured by the DPS, we ran an ANOVA (without bootstrapping) including the two independent variables priming and games and the dependent variable DPS. Our analysis did not show any significant interaction effect between the two independent variables (*p* > 0.05); hence, we ran separate analyses: one for priming and another for game, investigating the main effects.

The analysis was not able to identify any significant effect of the independent variables on the DPS (*p_priming_* > 0.05; *p_games_* > 0.05). We can conclude on the basis of the given statistical power that within our test, the CCMG and the priming did not affect social inclusion as measured by the DPS.

##### Hypothesis 2: Awareness of ingroup bias mediates the effects of the CCMG on social inclusion

To investigate if awareness mediates the effects of the CCMG on our social inclusion measures, we ran a mediation analysis (Hayes, 2016) with 5,000 bootstrap samples for each dependent variable (aggregated distance estimations, DPS) separately, with the two awareness questions (general awareness and awareness games) as mediators. We did not find any significant mediation effects for the distance measure or the DPS (p > 0.05).

##### Hypothesis 3: Playing the CCMG decreases explicit cultural bias

To investigate if playing the CCMG decreases explicit cultural bias, we ran a MANOVA (without bootstrapping) with the two independent variables priming and games, and the dependent variables AAsS, ATMA, ExpAsians, and ExpArabs, to investigate if there was an interaction effect between the two independent variables priming and games. We did not find a significant interaction effect (*p* > 0.05). We then ran MANOVAs for the two independent variables priming and games separately, with the explicit measures as dependent variables to test our hypothesis. We did not find significant main effects for priming or games (*p_priming_* > 0.05; *p_games_* > 0.05). Hence, we were unable to reject the null hypothesis and conclude that the CCMG and the priming did not affect cultural bias as measured by our explicit measures.

##### Hypothesis 4: Awareness of ingroup bias mediates the effects of the CCMG on explicit cultural bias

To investigate potential main effects of the independent variables on the awareness of ingroup bias, we calculated ANOVAs (without bootstrapping) with the priming condition and the game condition as independent variables and the awareness questions as dependent variables. We did not find any significant interaction effects or main effects of the independent variables on the awareness level (*p* > 0.05).

To investigate if awareness mediates the effects of the CCMG on our dependent variables, we ran a mediation analysis (Hayes, 2016) with 5,000 bootstrap samples for each dependent variable (AAsS, ATMA, ExpAsians, ExpArabs) separately, with the two awareness questions (general awareness and awareness games) as mediators. We did not find any significant mediation effects for the cultural bias measures (*p* > 0.05).

### Discussion

In line with the results of Study 1, we found that playing the CCMG increases social closeness as measured by our distance estimation task. Hence, this study validates the results of Study 1 with a different sample (Caucasian Australians vs. US-Americans). Adding a second control game to our Study 2, we found that playing the CCMG does not only increase social closeness in comparison to a control game only presenting the ingroup (i.e., Western culture) but that it also increases social closeness in contrast to being exposed to the different cultures. Hence, the effects of the CCMG on social closeness cannot be explained solely by the exposure effect but support the benefits of a dual identity.

However, playing the CCMG did not decrease explicit bias or increase explicit inclusion as measured by our questionnaires. There are some possible explanations for the discrepancy between the results found when using explicit and implicit attitude measures. The distance task as a measure of implicit attitudes might be picking up a different concept than our other, explicit measures. Our experimental design might have led to our intervention working on a more implicit than explicit level. We did neither explicitly introduce the CCMG to our participants as a bias reduction intervention nor explicitly placed the experiment in an intercultural context in any other way. This may have prevented participants from processing the CCMG consciously, hence only affecting implicit attitudes rather than explicit ones.

In contrast to classical intercultural interventions, we did not explicitly mention the multi-cultural context of our study as to not influence the participants in answering in a socially desirable way. However, this might have prevented the participants from actually becoming aware of their own cultural biases. This might be the reason we did not find any effects of the CCMG on awareness or any mediating effects of awareness on the relation between the games and the dependent variables. The most suitable framing of the CCMG to optimise its positive effects should be investigated further.

## General discussion

Research around diversity in general and cultural diversity specifically oftentimes focuses on reducing ingroup bias (Shore et al., 2011). Indeed, to this day, interventions are still trialled focusing on stereotypes and reducing biases (e.g., Ozaydin et al., 2021; Sarı and Alkar, 2022). However, only reducing bias may not be enough to foster the full benefits of a diverse society and a workplace employing diversity. An emerging stream of literature highlights the importance of social inclusion as key to increase the positive effects of diversity, such as innovativeness and creativity (Shore et al., 2011, 2018). In this research, we contribute by assessing how an application of the DIM influences social inclusion in a multicultural setting. Our findings indicate that encouraging people to adopt a dual identity perspective can increase the perceived closeness toward other cultures. This implies that people are more likely to identify an individual from a different cultural background as being similar to themselves (Trope et al., 2007) and is linked to greater liking (Swift, 1999) of former outgroup members, now ingroup members.

In our present research, we extend the current literature by showing how the DIM and the use of active categorization can be adapted in the area of social inclusion. Our approach based on the DIM aims to broaden the positive effects of ingroup bias to individuals formerly perceived as outgroup members, While still making them feel valued in their own individuality (Dovidio et al., 1998). We developed the CCMG to induce a dual identification and increase closeness between different cultures. This effect was reliable across two countries and presents preliminary support for the benefits of a social inclusion intervention inspired by the DIM. Effective interaction between people from different (cultural) backgrounds is essential in today’s globalized world, and greater degrees of subjective closeness can make the interaction easier and more effective (Swift, 1999). Our findings are in line with the backbone idea of DIM in that when their needs for uniqueness and belonging are met simultaneously, people are able to work to their full potential (Ferdman and Sagiv, 2012).

Traditional DIM interventions mainly prime a dual identity through a once-off passive categorization (e.g., Glasford and Dovidio, 2011; Charnysh et al., 2015). This does seem to work for the duration of the studies; however, our approach involves the participants in an active categorization process. As shown in diverse streams of literature, active involvement can strengthen a manipulation and is appreciated by participants (e.g., Albarracín et al., 2005; Markant et al., 2016; Chen et al., 2018). Our approach uses game design elements to increase the active involvement of participants and to induce fun, hence strengthening the motivation of participants and the desired impact of the manipulation (Albarracín et al., 2005; Muntean, 2011; Deterding, 2012; Schoech et al., 2013; Ramírez Galleguillos et al., 2021). The CCMG requires participants to actively categorize every single tile into groups and is played more than just once. The frequent, active categorization and the fun it evokes, should strengthen the manipulation, induce more effective learning, and might enable potential long-term effects (e.g., Schou, 1985; Kvam, 2000; Veenhof et al., 2006; Ramírez Galleguillos et al., 2021). As the game encourages participants to use certain categories a multitude of times during the game, this should influence the salience of said categories. Indeed, our results show that superordinate categories became more salient in our grouping task. Encouraging participants to use the categories in the memory game might influence their categorization in general. This may support a long-term effect as the general (social) categorization process can be influenced by chronic use of certain categories (Hornsey, 2008). Further studies should investigate this potential benefit of our approach in longitudinal study designs. However, even if the CCMG does not show lasting effects after one or two sessions, the gamified nature of the intervention and the fun it evokes can encourage people to keep on playing the game, making it more likely that the individuals benefit from the actively induced dual identification long-term compared to the more passive approaches to inducing DIM.

Although we believe that our application for DIM is particularly suitable to harness social inclusion, we did expect that playing the CCMG could reduce explicit bias – which we did not find in our experiment. A reason for this lack of effect may be the result of the nature of our application of DIM. Our approach targets basic perception and cognitive mechanisms, as it (1) works on a visual level, and (2) encourages active social Categorization. Visual perception, as well as categorization, are automated processes that happen largely outside of an individual’s awareness (Mervis and Rosch, 1981; Velmans, 1999; Kawakami et al., 2017). In this way, our intervention seems to specifically target *implicit* attitudes (see Kawakami et al., 2017). Indeed, we understand the distance task as a measure of implicit attitudes. This measure has been used and validated in different contexts (e.g., Carbon and Leder, 2005; Carbon, 2010; Carbon and Hesslinger, 2013). To understand the meaning of the distance task better, future research should investigate the relationship between the distance task and other implicit measures. Our intervention affected the implicit attitudes, but we did not find an effect on explicit measures. Hence, including alternative implicit measures in a next study can help us gain more insights about which processes underlie a dual identification and which mechanisms influence the change in attitudes that we recorded in this research. Further, what these results show is that while DIM may be beneficial to induce implicit inclusion, it does not extend to explicit bias. Indeed, to harness diversity, both are required and thus the CCMG cannot be deemed a solve-it-all. While these results on explicit bias and explicit awareness are disappointing, these results do provide valuable insights into the nuanced nature of the CCMGs effectiveness. Indeed, further research can address how an extension or addition to the CCMG might achieve both implicit social inclusion attitudes, as well as explicit attitudes such as to maximise behavioural effectiveness.

In this present project, we did not measure the level of dual identification in a direct way. For once, measuring dual identification is difficult, see Appendix O for further information. However, the more influential reason for our decision not to measure dual identification is a theoretical one. Traditionally, the goal of interventions in the DIM research is to prime a dual identification in the participants themselves. This can be achieved by letting the participants read about the benefits of a dual identity or through introducing visual cues (González and Brown, 2003; Glasford and Dovidio, 2011; Verkuyten, 2017). The CCMG also employs visual cues; however, the CCMG was developed to encourage players to break out of their usual thinking patterns and perceive society as a unified overarching society representing a broad variety of cultures and diverse individuals. Hence, rather than changing their own self-identification, the CCMG specifically focuses on other-identification into ones already existing dual identification. The recategorization on a superordinate level aims to make people perceive the other person as part of their own group, which is why the CCMG is expected to make the players’ perception salient that others belong to a group with which the players already identify (i.e., human). It accentuates the similarity to other ingroup members (Turner and Reynolds, 2001) and can thus change one’s appreciation of others. Differences in values and norms between individuals and, as such, contrasting worldviews are an influential form of dissimilarity that might even play a bigger role than the actual cultural categories (Havermans and Verkuyten, 2021). The CCMG requires players to categorize images based on similarities. This focus on similarities is expected to reduce the perceived dissimilarity between the cultures, assimilating the various cultural worldviews. Arts and community-based intercultural interventions are similar in this approach. The tenet here is that arts and aesthetics allow for a common ‘language’ to come to the foreground where differences take a back seat and the focus is on commonalities and positive intercultural experiences altering one’s view of others (Catalano and Morales, 2022). Future research should explore the effects of the CCMG on the worldview of the players and their perception of others directly. Just as assigning individuals to intercultural groups, introducing participants to such groups in the CCMG could change the way the players construe cultural groups, affecting the perceptual and evaluative processes relevant in a given situation (van Bavel and Cunningham, 2010).

Theory suggests that being (visually) exposed to certain stimuli repeatedly does generally lead to a more positive evaluation of said stimuli (mere exposure effect; Zajonc, 1968, 2001). However, the game does not seem to work through a mere exposure effect as distinguishable from the induction of a dual identity. When we implemented a control game that exposed participants to all cultures but did not have them actively be categorised together, we did not see an increase in social closeness compared to the control. Although people are not explicitly aware of this process, the visual nature of the game might still increase participants’ attention towards other cultures in their daily lives. Being exposed to stimuli can increase their salience in someone’s mind, making those stimuli more available (e.g., Chen and Li, 2009). Salience can shift peoples’ attention toward these contents/issues (e.g., Bordalo et al., 2012). Attentional processes play an essential role in information processing as they take effect in early stages and can influence the following stages, such as interpretation and evaluation (Tuschen-Caffier et al., 2016). Therefore, the game may influence categorization in future situations mimicking the visual type of categorization that occurred during playing the game.

So far, we have looked at attitudes, not at behavior. We are ultimately interested in how dual identification through an active categorization can change behavior and potentially facilitate a socially inclusive work environment. However, cognitive changes—such as the changes in cultural distance—are attenuated through different mediators like motivational and action-oriented processes before they finally result in behavior (Sniehotta, 2009; Michie and Johnston, 2012); this is why it is not necessarily sufficient to create knowledge structures or intentions for behavior change to induce the desired changes (Contento et al., 1995). Hence, future research should investigate the effects of the CCMG on actual behavior.

We conducted the experiments with samples from two different countries, presenting first supporting evidence for the generalizability of the CCMG. However, both of those countries are part of the Western culture. Future research should investigate the effects of the CCMG on samples from the other cultures included in the game (i.e., Arabs/Middle Eastern). Additionally, it might be beneficial to test if the effects of the CCMG on social closeness are of a more general nature than tested so far. Even though the CCMG directly targets the three cultures included in the game, there might be spill-over effects on the relationship with other cultures as well.

We believe that our game can be adopted as a bias training material focused on increasing inclusion, in addition to existing bias training that is usually focused on the reduction of discrimination. Social inclusion is an ongoing issue; its scope is only strengthened in times of globalization. But the workplace is not the only context in which our intervention could be applied. Thanks to the gamification techniques used and the fun our game evokes, we believe our intervention may be beneficial in the work with children. Targeting implicit attitudes toward cultural inclusion from a young age may be especially promising because implicit attitudes are learned (Dovidio et al., 2013). Automaticity develops through repeated occurrence, practice, and ultimately overlearning (Wyer and Hamilton, 1998; Dovidio et al., 2013). Hence, children could benefit from the game specifically as their implicit attitudes might not be as ingrained in their cognition in comparison to adults. Next to our game, other types of applications based on our approach could be beneficial. For instance, children at school could be instructed to play Minecraft together in a shared realm/world which aims to facilitate collaboration and inclusion by incorporating visual representations or cues for the superordinate groups class or school and encourages the categorization of diverse classmates into one of the superordinate groups.

## Conclusion

Reducing cultural barriers helps to achieve greater degrees of subjective closeness, making the interaction easier and more effective (Swift, 1999). Our approach draws together insights from different areas of psychology and gamification, contributing to the understanding and cooperation between those fields as a basis to create societal impact. This paper presents first results indicating that our approach of actively inducing a dual cultural identity could help to bring people in a globalized world closer together. Active dual identification does not need to be limited to the cultural background; instead, it can potentially be applied to a wide range of social groups, such as gender, age, or sexual orientation and hence could facilitate the encounter between individuals from different backgrounds in a wide range of social situations.

## Author’s note

The data that support the findings of this study are available on request from the corresponding author, JP. The data are not publicly available due to conditions associated with our human ethics approval.

## Data availability statement

The raw data supporting the conclusions of this article will be made available by the authors, without undue reservation.

## Ethics statement

The studies involving human participants were reviewed and approved by RMIT University Business & Law College Human Ethics Advisory Network. Written informed consent for participation was not required for this study in accordance with the national legislation and the institutional requirements.

## Author contributions

JP conducted the experiments and wrote the first draft of the manuscript. JP and JB performed the statistical analysis. JP, AN, C-CC, JS, and JB contributed to the conception and design of the study. All authors contributed to the article and approved the submitted version.

## Conflict of interest

The authors declare that the research was conducted in the absence of any commercial or financial relationships that could be construed as a potential conflict of interest.

## Publisher’s note

All claims expressed in this article are solely those of the authors and do not necessarily represent those of their affiliated organizations, or those of the publisher, the editors and the reviewers. Any product that may be evaluated in this article, or claim that may be made by its manufacturer, is not guaranteed or endorsed by the publisher.

## Footnotes

^1^ http://www.web-games-online.com/memory/

^2^ https://fromabcstoacts.com/multicultural-matching-game/

^3^Important note: The game includes images depicting stereotypical items or scenes from each culture. The implementation of such features often associated with these cultures aims to enable participants to clearly distinguish between the different cultures as a way to create an intervention targeting and facilitating intercultural relationships. We do not want to create the impression that the selected images represent the most stereotypical scenes or items for the associated cultures, nor that these images fully capture the variability and complexity of these cultures (e.g., not everyone from a Western background is of Christian faith).
