# Supplementary material for: An application of the dual identity model and active categorization to increase intercultural closeness
Source: Front Psychol. 2022 Sep 13;13:705858. doi: 10.3389/fpsyg.2022.705858 (PMC9514141; doi:10.3389/fpsyg.2022.705858)
Supplement: Supplementary file 1 [file Data_Sheet_1.pdf]

Supplementary Material

Appendix A

Information about the general procedure of maximising the statistical power of the experiments and the inclusiveness of the samples

We sought to maximise the statistical power of our experiments in various ways. We conducted a priori power analyses for all our studies, calculating the necessary sample sizes. Where appropriate, we included control conditions, manipulation checks, and covariates in our experiments. We randomly assigned the participants to the different manipulations. Where our dependent variables were measured via questionnaires, we ran factor analyses and reliability analyses to explore if the used questionnaires were reliable with our sample and excluded individual items from the resulting aggregate measures to increase the internal consistency of our measures. The exact procedures are described in the methods sections of each experiment or the corresponding appendices.

This research follows the Australian National Statement on Ethical Conduct in Human Research and as such aims to be inclusive in the samples it has recruited. Where we did have to sample specific groups in order to test our hypotheses effectively, we sought additional ethics approval. Our samples were collected in two countries to increase the generalisability of our results and are representative for each population in terms of age, gender, and education level. More details about the samples can be found in the methods sections of each experiment.

Appendix B

Kimchi-Palmer-Figures task (Kimchi and Palmer, 1982)

This task contains visual target figures in which small geometrical shapes (e.g., a triangle) form a larger geometrical shape (e.g., a square; see **Supplementary Figure 1** below). The participants are asked to indicate which one out of two selection options resembles the target more closely: 1) the figure, where the local elements match the global element of the target (abstract construal) or 2) the figure, where the local elements match the local elements of the target figure (concrete construal; Burgoon et al., 2013). The selection options were dummy coded (0 = concrete, 1 = abstract), added up over the 16 targets and divided through 16, leaving us with results between 0 and 1 (0 = participant chose the concrete figure in all 16 cases; 1 = participant chose the abstract figure in all 16 cases). We expected the participants in the abstract level condition to choose the abstract Kimchi-Palmer figure more often than the concrete ones and to use fewer categories than in the concrete level condition and vice versa.

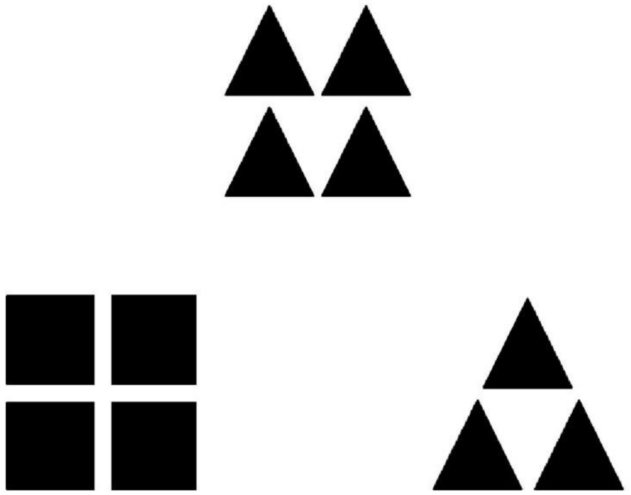

Supplementary Figure 1. Example of one set of the Kimchi-Palmer-Figures task.

Appendix C

Abstraction level of the categories used in the abstraction task/manipulation check

Terms the participants used to label the categories they used while playing the games in the pre-test.

Category	Concrete = 1	2	3	Abstract = 4
**Food**	**Dish**: (Ham/Cheese)Burger, Sandwiches, Sushi		**Food Category**: Fast food, American food, food wraps, junk food, healthy food	**Food/eating/nutrition:** Food, meals, protein, dinner, lunch, savory, groceries, entrees, international/exotic food, merienda (afternoon snack), breakfast, main course, meats, veggies, foreign food, circle, take out food
**Sweet bakery**	**Named sweet pastry**: Doughnuts, mooncake		**Baked goods**: Sweet bakery, cake, breads, cookies, biscuits	**Named food category** desserts, candy, snacks, sugarfood, pastries, baked (goods), (sweet) food, sweet treats, sweet(s)
**Landscapes**	**Elements depicted in the images**: Fields, roads, grass(y), (blue) sky, pastures, ocean, sandy places, islands, paths, farm land, hay field, mowed fields, grass lands, farm, lawn, trails, flat fields, beach, sands, water	**Elements with associations:** open grass fields		**Holistic impression**: Land(scape), destination, scenery, open area, outdoors, nature, country, vacations, biomes, isolation, settings, open fields, horizon, plains, ground, wilderness, outside, geography, naturescape, desolate area, places, terrain, long view, paradise, tourist place, wide open spaces
**Houses of prayer**	**Named houses of worship, their parts**: churches, cathedrals, tower, temples, buildings with spires, spires	**Named buildings:** Churches and castles	**Associated buildings:** Historical buildings, religious buildings, national buildings	**Non-named instances of buildings or higher-level associations:** Houses of worship, monuments, (big, nice, important, single, large, fancy) buildings, shrines, religion, culture, castles, palace, mansions, landmarks, houses, large architecture, places

**Words**	**Named meaning of the words**: (mental) words, sayings, mind/memory/remember, mind states, remembering, memories			**Context of the words**: Languages, writing, letters, white lettering, text, brain, symbols, memory in different languages, threes, (white) cards, country, written/ white words
**Girls**	**Physical appearance:** Blonde women, models, pretty girls, cuties, beautiful woman, blond ladies, pretty (woman), young woman	**Individual**: Single, only, single people, single woman, girl alone, woman standing alone, individual woman, one girl, single girl, alone	**Gender**: Girls, females, woman, ladies, blonde, female photo, female portrait, chicks	**Human beings or associations:** person, people, individual, (one) person, portraits, (model) photos, pictures, raza (race), woman of culture, happiness, human, selfie
**Wedding couples**	**Focus on the/one individual**: Bride & Groom, bridal, brides	**Focus on couples at their wedding**: Wedding couples, married, newlyweds, married couples	**Focus on whole day/ceremony**: Weddings, marriage, wedding day, wedding pictures, marriage ceremony photos	**Concept of being together:** partnership, taken, events, match, parents, love, groups of people, couples
**Villages/ towns**	**Named types of residences**: Condos, apartments, houses, seaside village, townhome, condominium, apartment buildings, village buildings, china homes, city buildings	**Named collection of houses/ types of habitats**: towns, villages, city, neighbourhood, suburbs	**Collection of buildings:** houses in diff countries, multiple buildings	**Concept of living:** Homes, housing, habitats, skyline, property, places, living spaces, civilisation, residences, community, residential area, urban, abode, living quarters

Missings: Pizza, match, white, test, camping, past

Appendix D

Distance task USA sample

The participants are presented with the following city pairs in a random order and are asked to *estimate* the distances between the presented cities in miles.

BE = Beijing
HK = Hongkong
LA = Los Angeles
NYC = New York
BG = Baghdad
KB = Kabul

1. BE – HK
2. LA – NYC
3. BG – KA
4. HK – LA
5. NYC – BG
6. BE – LA
7. HK – NYC
8. BE – KA
9. LA – BG
10. BE – NYC
11. HK – BG
12. LA – KA
13. BE – BG
14. NYC – KA
15. HK – KA

Appendix E

Categorisation Task

Images of the categorisation task in Study 1. Images reproduced from www.pixabay.com and www.pexels.com.

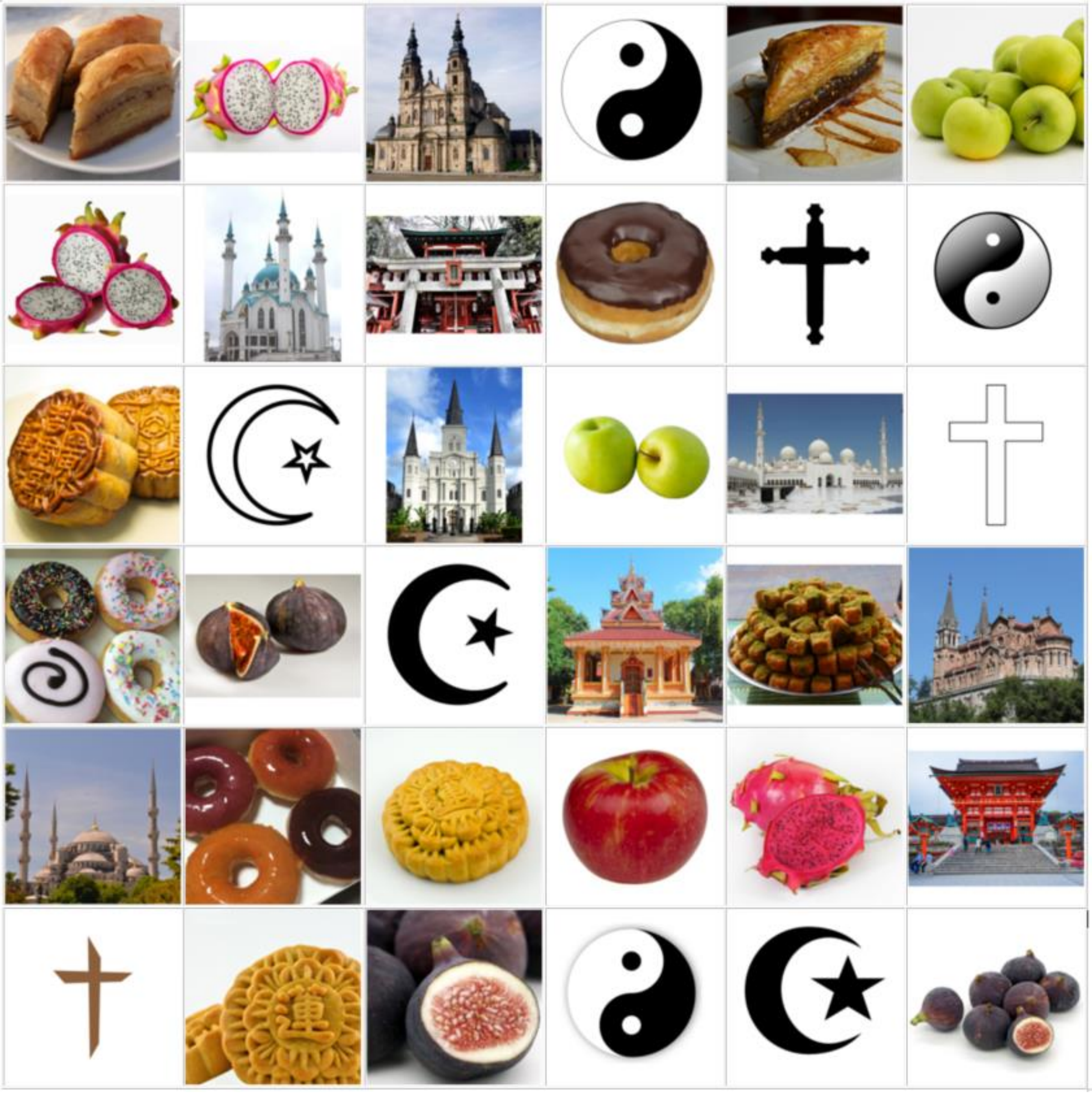

Appendix F

Priming tasks & Non-priming tasks

Priming tasks:

There are various ways of priming cultural background. For example, asking for cultural background can in itself work as a prime for cultural identity (e.g., Kirnan et al., 2009). We included the following three questions related to one's own cultural background in our study:

Demographic questions concerning cultural background

1) In which country were you born?
2) What is your nationality? Please indicate if you have more than one nationality and specify them.
3) What is your country of residence/citizenship?

In line with this argumentation, questionnaires about (cultural) group identification can be used as priming tools as well (e.g., Williams et al., 2008). We decided to include the two subscales *membership* and *identity* from the Collective Self-Esteem scale (CSE; Luhtanen and Crocker, 1992) as used by Verkuyten (2008) in our study:

Group identification questionnaire

Below you will find a list of statements. Please read each statement carefully and decide if that statement describes you or not. Please indicate your agreement from 1 = *strongly disagree*, to 7 = *strongly agree*. *Reverse-coded items are marked with (r).*

Membership Scale:

1. I am a worthy member of the ethnic group I belong to.
2. I feel I don't have much to offer to the ethnic group I belong to. (r)
3. I am a cooperative participant in the ethnic group I belong to.
4. I often feel I'm a useless member of my ethnic group. (r)

Identity Scale:

5. Overall, my ethnic group membership has very little to do with how I feel about myself. (r)
6. The ethnic group I belong to is an important reflection of who I am.
7. The ethnic group I belong to is unimportant to my sense of what kind of a person I am. (r)
8. In general, belonging to my ethnic group is an important part of my self-image.

A method that is not specific to cultural background but widely used in priming studies is storytelling (e.g., Dijksterhuis and van Knippenberg, 1998; Sassenberg and Moskowitz, 2005). Participants are asked to talk or write about their experiences associated with the concept to prime. We could not identify such a method specifically designed for priming the Australian culture, but we adapted the procedure for our context. In our study, we asked the participants to describe an experience that made them proud to be Australian using the following instructions:

Essay writing

In this task, we ask you to write a short essay.

> Please think about a (recent) event that made you proud to be Australian. Write a short essay about your experience. Go in as much detail as you can, but please limit yourself to approximately 5 minutes of writing. You will be able to go on to the next question after a couple of minutes. After 5:30 minutes, the survey will automatically go on to the next question.

Non-priming tasks:

Following the structure of our priming tasks, participants in the non-priming condition were presented with three tasks as well.

Personal questions unrelated to cultural background

1) Are you right-handed, left-handed or both-handed?
2) Do you wear glasses or contact lenses?
3) Which supermarket do you prefer?

Second, we presented the two subscales *past focus* and *future focus* of the Temporal Focus Scale (TFS; Shipp et al., 2009):

Temporal Focus Scale

Past Focus:

1. I replay my memories of the past in my mind.
2. I reflect on what has happened in my life.
3. I think about things from my past.
4. I think back to my earlier days.

Future Focus

1. I think about what my future has in store.
2. I think about times to come.
3. I focus on my future.
4. I imagine what tomorrow will bring for me.

Third, we asked the participants to write an essay about their last grocery shopping experience with the following instructions:

Essay writing

> In this task, we ask you to write a short essay.
>
> Please think about your last grocery shopping. Write a short essay about your experience. Go in as much detail as you can, but please limit yourself to approximately 5 minutes of writing. You will be able to go on to the next question after a couple of minutes. After 5:30 minutes, the survey will automatically go on to the next question.

Appendix G

Distance task Australian sample

The participants are presented with the following city pairs in a random order and are asked to *estimate* the distances between the presented cities in km

SY = Sydney
PE = Perth
BE = Beijing
HK = Hongkong
BG = Baghdad
KB = Kabul

1. BE – HK
2. SY – PE
3. BG – KA
4. HK – PE
5. SY – BG
6. BE – PE
7. HK – SY
8. BE – KA
9. PE – BG
10. BE – SY
11. HK – BG
12. PE – KA
13. BE – BG
14. SY – KA
15. HK – KA

Appendix H

Personal Dimension of the Diversity Perceptions Scale (DPS; Mor Barak et al., 1998)

Below you will find a list of statements. Please read each statement carefully and indicate your agreement from 1 = *strongly disagree*, to 7 = *strongly agree. Reverse-coded items are marked with (r).*

Personal diversity factor:

1. Knowing more about cultural norms of diverse groups would help me be more effective in my job.
2. I think that diverse viewpoints add value.
3. I believe diversity is a strategic business issue.

Personal comfort factor:

4. I feel at ease with people from backgrounds other than my own.
5. I am afraid to disagree with members of other groups for fear of being called prejudiced. (r)
6. Diversity issues keep some work teams here from performing to their maximum effectiveness. (r)

Appendix I

Attitudes to Asians Scale (AAsS; Walker, 1994)

Below you will find a list of statements. Please read each statement carefully and indicate your agreement from 1 = *strongly disagree*, to 7 = *strongly agree*. *Reverse-coded items are marked with (r).*

1. Allowing Asians to immigrate to Australia benefits Australian society. (r)
2. Australia should aim at closer contact with Asian countries. (r)
3. More Asians should be allowed to migrate to Australia. (r)
4. I would not like an Asian to be my boss.
5. Asian migrants are as friendly as people born in Australia. (r)
6. Asian and white people just don't mix well.
7. Australia must be very careful not to let too many Asians into the country or they'll take over the place.
8. One trouble with Asian business people in Australia is that they stick together and prevent other people having a fair chance in competition.
9. I wouldn't like any member of my family to marry an Asian immigrant.
10. I don't like Asians.
11. Asians are very productive people and should be allowed to settle in Australia. (r)

Appendix J

Attitudes Toward Muslim Australians (ATMA; Griffiths and Pedersen, 2009)

Below you will find a list of statements. Please read each statement carefully and indicate your agreement from 1 = *strongly disagree*, to 7 = *strongly agree*. *Reverse-coded items are marked with (r).*

1. The average Arab is as reasonable as everyone else. (r)
2. Islamic schools should not be allowed in this country.
3. Arabs do not respect freedom of speech.
4. Islam is no threat to Australia's freedom. (r)
5. All Arabs are potentially terrorists.
6. The majority of Arabs are law abiding citizens. (r)
7. The Arabs are a peace loving community. (r)
8. Islam is threatening Australia's freedom.
9. Arabs are respectful and sensitive toward other religions within Australia. (r)
10. Arabs have a hatred of western values.
11. Islam is a dangerous religion and should be banned in Australia.
12. As a multi-cultural nation, Australians should accept that Arabs are entitled to express their religious identity freely. (r)
13. Islamic beliefs and customs are not compatible with multicultural Australia.
14. I do not want my family mixing with Arab families.
15. Arabs do not want to obey our laws.
16. Arabs are just as friendly as other Australians. (r)

Appendix K

Covariates and their measurement

The literature suggests several possible influence factors on cultural ingroup bias, with the most prominent being age (e.g., Griffiths and Pedersen, 2009), gender (e.g., Sidanius et al., 2000; Griffiths and Pedersen, 2009), education level (e.g., Wagner and Zick, 1995; Griffiths and Pedersen, 2009; Pedersen and Hartley, 2012), political orientation (e.g., Griffiths and Pedersen, 2009; Pedersen and Hartley, 2012), mood (e.g., Forgas and Fiedler, 1996), and self-esteem (e.g., Lönnqvist et al., 2015). Age, gender, and education level were measured with single items within the demographic questions. Additionally, all participants were asked with which political party they identify most (Liberal Party, Labour Party, Greens), and to fill in measures for self-esteem (Rosenberg Self-Esteem Scale, RSES; Rosenberg, 1979) and mood (Mood Short Form, MSF; Peterson and Sauber, 1983). The full questionnaires measuring self-esteem and mood can be found below. *Reverse-coded items are marked with (r).*

Rosenberg Self-Esteem Scale (RSES; Rosenberg, 1979)

1. On the whole, I am satisfied with myself.
2. At times I think I am no good at all. (r)
3. I feel that I have a number of good qualities.
4. I am able to do things as well as most other people.
5. I feel I do not have much to be proud of. (r)
6. I certainly feel useless at times. (r)
7. I feel that I'm a person of worth.
8. I wish I could have more respect for myself. (r)
9. All in all, I am inclined to think that I am a failure. (r)
10. I take a positive attitude toward myself.

Mood Short Form (MSF; Peterson and Sauber, 1983)

1. Currently, I am in a good mood.
2. As I answer these questions I feel cheerful.
3. For some reason I am not very comfortable right now. (r)
4. At this moment I feel edgy or irritable. (r)

Appendix L

Social Desirability Scale-17 (SDS; Stöber, 2001)

Below you will find a list of statements. Please read each statement carefully and decide if that statement describes you or not. If it describes you, check the word *'true'*; if not, check the word *'false'*. *Reverse-coded items are marked with (r).*

1. I sometimes litter. (r)
2. I always admit my mistakes openly and face the potential negative consequences.
3. In traffic I am always polite and considerate of others.
4. I always accept others' opinions, even when they don't agree with my own.
5. I take out my bad moods on others now and then. (r)
6. There has been an occasion when I took advantage of someone else. (r)
7. In conversations I always listen attentively and let others finish their sentences.
8. I never hesitate to help someone in case of emergency.
9. When I have made a promise, I keep it – no ifs, ands or buts.
10. I occasionally speak badly of others behind their back. (r)
11. I would never live off other people.
12. I always stay friendly and courteous with other people, even when I am stressed out.
13. During arguments I always stay objective and matter-of-fact.
14. There has been at least one occasion when I failed to return an item that I borrowed. (r)
15. I always eat a healthy diet.
16. Sometimes I only help because I expect something in return. (r)
17. I have tried illegal drugs (for example, marijuana, cocaine, etc.). (r)

Appendix M

Data preparation Study 2

As mentioned in the body text of the paper, we ran principal component analyses (PCAs) to check for the appropriateness of the scales (DPS, ATMA, AAsS, MSF, RSES, SDS). Additionally, we calculated Cohen's alpha as a reliability check for the questionnaires. The PCAs for the AAsS, ATMA, MSF, and RSES validated our use of these scales, and we obtained an acceptable Cohen's alpha for these scales (α_{AAsS} = .926; α_{ATMA} = .962; α_{MSF} = .815; α_{RSES} = .905; α_{CSE} = .823). However, for the SDS, we decided to exclude the two items "I sometimes litter" and "I never hesitate to help someone in case of emergency", obtaining an acceptable internal consistency (α_{SDS} = .718). These items differ from the other ones as they describe an exceptional emotional situation (emergency) or dip into a rising environmental consciousness; their difference is supported by the fact that both items have factor loadings <.3.

The analyses for the DPS were not consistent with the literature; we did not identify the two-factor structure for the items (2 subscales with 3 items each) as suggested by the literature but found a two-factor structure based on statistical artefacts (the first factor representing the reverse coded items, the second factor representing the non-reverse coded items). Accordingly, we then ran a PCA forcing one factor, and results indicated us to exclude one item with a factor loading <.3 ("Diversity issues keep some work teams here from performing to their maximum effectiveness") from our analysis. This item is conceptually different as it asks participants to evaluate other work teams, whereas the other items target the participant's own beliefs and feelings about diversity. However, even after excluding the item, the internal consistency of the DPS was still poor (α_{DPS} = .587), and thus it is advised to interpret the results from the DPS very carefully.

Appendix N

Relationship between the dependent variables in Study 2

To investigate the relationship between our dependent variables, we ran Pearson correlations between all the dependent variables (explicit bias measures: ExpAsians, AAsS, ExpArabs, ATMA; social inclusion measure: DPS; social distance measure: aggregated distance estimations; see table below). All the explicit bias measures correlated positively with each other, with $r_{Pearson}$ ranging from .281 to .649. This indicates that if participants expressed explicit bias against one culture outside their own Western culture, they tended to be biased towards the other culture as well. The results for the explicit social inclusion measure show relatively small but significant negative correlations to the explicit bias measures with $r_{Pearson}$ ranging from -.211 to -.486. Participants who expressed positive attitudes towards diversity and social inclusion tended to express fewer negative attitudes toward other cultures. The distance measure shows very small to no correlations to the explicit bias and social inclusion measures, indicating (if at all) a reversed relationship between implicit and explicit attitudes in this study (higher levels of implicit closeness associated with lower levels of explicit inclusion and with higher levels of explicit bias). This is important to note specifically in light of the results we present below.

Pearson correlation between the dependent variables.

	**Distances**	**Exp Asians**	**AAsS**	**Exp Arabs**	**ATMA**	**DPS**
**Distances**	-					
**ExpAsians**	-.034	-				
**AAsS**	-.133*	.497**	-			
**ExpArabs**	-.066	.505**	.281**	-		
**ATMA**	-.134*	.435**	.649**	.601**	-	
**DPS**	.142*	-.322**	-.486**	-.211**	-.436**	-

*** $p<0.001$, ** $p<0.01$, * $p<0.05$.

Appendix O

Difficulties in the measurement of dual identification

In contrast to most DIM studies, the targeted superordinate category in our experiments is a general human or global social identity. Most social identity literature focuses on more concrete identities in terms of comparison between various social groups instead of the human identity as highest level of self-categorisation (Grimalda et al., 2018). Studies that do measure this specific human identification usually do so using items solely focusing on one level of identity at a time, typically not targeting complex identity patterns (e.g., McFarland et al., 2012; Grimalda et al., 2018). Some authors argue that a measure assessing individual levels of identification cannot reflect a dual identity. As Martinovic and Verkuyten (2014) put it, a "[d]ual identity does not refer to one's thoughts and feelings about each constituent group separately, like two component identifications that are subsequently combined by a multiplicative product term in statistical analysis" (p. 714). Rather, the authors conceptualise a dual identity as a new category that is distinct from the original individual ones. However, the measures we identified in our literature review that assess dual identity following this conceptualisation typically target more concrete identities than a human identity (e.g., the host national category; Martinovic and Verkuyten, 2014) and are, unfortunately, not easily adaptable to the more abstract superordinate categorisation of all mankind. Hence, we decided to focus on experiments with carefully designed control games and their effects on our dependent variables and basic categorisation processes as indicators for induced dual identification.
